# Screening of *Spirulina* strains for high copper adsorption capacity through Fourier transform infrared spectroscopy

**DOI:** 10.3389/fmicb.2022.952597

**Published:** 2022-07-29

**Authors:** Jinghua Liu, Changwei Zhu, Zhengpeng Li, Haoyuan Zhou

**Affiliations:** ^1^College of Agriculture and Bioengineering, Heze University, Heze, China; ^2^College of Life and Health Science, Anhui Science and Technology University, Fengyang, China; ^3^School of Public Administration, Shandong Technology and Business University, Yantai, China

**Keywords:** *Spirulina*, copper adsorption, FT-IR spectroscopy, principal component analysis, screening

## Abstract

Microalgae have emerged as promising biosorbents for the removal of toxic metals from industrial effluents due to the presence of various free functional groups. While the constitutes are distinct among different algal strains, it needs to screen the algae with high adsorption capacities for heavy metal ions by analyzing the algal components. In this study, a rapid and nondestructive Fourier transform infrared (FTIR) method combined PCA algorithm was used to discriminate algal strains according to their cellular components. With FTIR spectroscopy, we have found that the algal strains for high copper adsorption capacity (RH44, XS58, AH53, and RZ22) can be well differentiated from other strains *via* assessing the components involved in the biosorption of copper ions at the spectral window range of 1,200–900 cm^−1^ mainly attributed to polysaccharides. Correspondingly, the copper removal efficiency by different *Spirulina* strains was also measured by biochemical assay and scanning electron microscopy (SEM) in order to confirm the screening result. Compared with the chemical measurement, the assessment based on spectral features appears fairly good in the evaluation and differentiation of copper adsorption capacity in various *Spirulina* strains. This study illustrates that FTIR spectroscopy may serve as a fast and effective tool to investigate the functional groups for copper ions binding in the *Spirulina* cell and it even offers a useful and accurate new approach to rapidly assess potential adsorbents for the high capacity of copper adsorption.

## Introduction

Heavy metal effluents produced from the industries, such as mining, electroplating, metal finishing, manufacturing, and textiles, impose a great threat to the environment due to their toxicity and nonbiodegradability. Therefore, removing heavy metal pollutants from wastewaters has become an increasing concern. Conventional methods applied to remove contaminants from wastewaters include coagulation, flotation, electrochemical removal, oxidation and reduction, ion exchange, membrane filtration, and reverse osmosis. However, it has been found that most of these techniques are either ineffective or not cost-effective in removing low concentrations of heavy metal ions from large volumes of wastewaters and they may even produce secondary wastes that require a further adequate disposal process (Ma et al., 2019; Zamora-Ledezma et al., 2021).

Biosorption of toxic metal ions by biological materials has been recognized as an economical and effective alternative to the existing methods for eliminating inorganic pollutants from industrial waste effluents (Ramírez Calderón et al., 2020; Sheikh et al., 2021). Algae, a kind of widely available biomaterials in many regions, are the potential adsorbents for their high metal binding capacities. It has been illustrated that the cell walls in the algae contain many phosphoryl, hydroxyl, carboxyl, sulfhydryl, and perhaps amino functional groups, which can provide binding sites for metallic ions (Li et al., 2019; Rajalakshmi et al., 2021; Ubando et al., 2021). In particular, *Spirulina platensis*, a kind of photosynthetic prokaryotes, has been successfully employed as a biosorbent for various components on the cell wall surface involving polysaccharides, proteins, and lipids (Zinicovscaia et al., 2020). While the functional groups are diverse among different *Spirulina* strains, it needs to screen the algal strains with high binding affinity by analyzing their components.

Traditionally, measurements of microalgal constituents require cellular disruption and extraction with organic solvents, such as acetone and dimethyl sulfoxide. Then, the extracts were analyzed by liquid or gas chromatography. However, these conventional detection methods have significant disadvantages, such as sophisticated operations, expensive equipment, and monitoring systems. Therefore, it is necessary to develop a more effective technology for rapid and convenient microalgal analysis.

Fourier transform infrared (FTIR) spectroscopy mainly relies on the absorbance of radiation at molecular vibrational frequencies to analyze the cellular compositions and their concentrations. Compared with the traditional methods, it has the distinctive advantages of fast, easy, nondestructive, and multiplex measurements (De Girolamo et al., 2019; Su and Lee, 2020). Actually, this technique has shown a great potential for distinguishing and quantifying different cellular constituents in microalgal cells (e.g., lipids, proteins, carbohydrates, and carotenoids). For instance, Pelusi et al. used the FTIR technique to analyze the composition in *Emiliania huxleyi* and found that the absorption intensity at 962.5 cm^−1^ attributed to a trans-C=C vibration which can be used to quantify the alkenones in haptophyte algae (Pelusi et al., 2016). Moreover, FTIR spectroscopy based on the advantages of its fast and multicomponent analysis in combination with chemometrics can also identify, discriminate, or classify different microalgal strains (Cebi et al., 2017; Grace et al., 2022). It can even provide a nondestructive and high-throughput method to screen the lipids, β-carotene, or astaxanthin hyperproducing microalgal strains based on the infrared spectroscopic tool (Liu and Huang, 2016).

There are many different species of *Spirulina* and their adsorption capacities for the heavy metal ions were distinct. Hence, in this study, the physiological characteristics of nine *Spirulina* strains were considered, and the copper adsorption capacity of different algal strains was also investigated. Furthermore, FTIR spectroscopy was also employed to measure the various compositions in different *Spirulina* strains. FTIR spectra were subsequently analyzed by the methods of principal component analysis (PCA). In particular, we have found that the evaluation based on the spectroscopic approach is well consistent with the adsorption capacity measured by biochemical assay and scanning electron microscopy (SEM). As a result, it suggests that FTIR spectroscopy may serve as a promising tool to classify different algal cells with the assistance of a multivariate statistics approach, and it can also be utilized to identify the *Spirulina* strains for removing heavy metal ions from polluted aqueous solutions.

## Materials and methods

### Cultivation of algae

Nine species of *Spirulina* were cultivated in Zarrouk's medium at 31 ± 0.5°C under a cycle of 14 h light/10 h dark photoperiod at an irradiance of 50 μmol photons/(m^2^·s). The growth rate was determined by measuring the optical density value at 560 nm through a UV-vis spectrometer (UV-1800, SHIMADZU) according to the following formula (Shanthi et al., 2021):


$$\begin{array}{l} K=\;\ln\left(X_{2}/X_{1}\right)/\left(t_{2}-t_{1}\right) \end{array}$$

where *K* means the growth rate of algal strains, and *X*_1_ and *X*_2_ refer to cell optical densities at time intervals *t*_1_ and *t*_2_ (day).

### Measurement of carbohydrate content

The algal cells were treated with a solution of 6 mol/L HCl to destroy the algal cell walls and heated in boiling water to hydrolyze the cellular carbohydrate for 10 min, and then 6 mol/L NaOH was added to neutralize the solution. The carbohydrate content was determined according to the dinitrosalicylic acid (DNS) method (Miller, 1959).

### Adsorption experiments

*Spirulina* cells were harvested by centrifugation and washed with distilled water to remove the residual culture medium. Then, they were dried at 50°C till constant weight, ground into powder with agate mortar and pestle, and sieved to obtain fine particles of 125 μm followed by copper adsorption (Chen et al., 2010).

The uptake of copper ions was carried out in conical flasks containing 40 ml of the desired concentration at 25°C in a rotary shaker (150 rpm) for 24 h to ensure adsorption equilibrium. A final concentration of 1 g/L for algal biomass power was incubated with CuSO_4_ solution with different initial Cu (II) concentrations ranging from 5 up to 100 mg/L.

Samples were collected by centrifugation at 10,000 *g* for 10 min after the adsorption processes reached equilibrium. The residual copper ions in the supernatant were chemically determined at a wavelength of 545 nm using the acetaldehyde–bis(cyclohexanone) oxaldihydrazone (BCO) method (Kim et al., 2021). The detection limit of this method is 0.1 mg/L, which is lower than the concentrations of copper ions in our experiments.

### FTIR spectroscopy measurements

After reaching the stationary phase, 100 μl of washed algal cells were smeared onto the CaF_2_ windows for FTIR measurements and freeze-dried prior to spectral recording (Liu and Huang, 2016).

All the samples were detected by an FTIR spectrometer (ALPHA-T, Bruker). The spectra were recorded in transmission mode using a 4 cm^−1^ resolution, encompassing the mid-IR region from 4,000 to 900 cm^−1^, with 32 scans for FTIR spectra. At least 15 samples were measured in each algal strain.

### Multivariate statistical analysis

Spectral data analysis of the algal cells was carried out using the OPUS 6.5 software provided by Bruker. The raw spectra were preprocessed by baseline correction and vector normalization according to the guide for users outlined in the software manuals before being converted into second derivatives. To evaluate component variations in *Spirulina* strains, the FTIR spectra were truncated to the ranges of 3,100–2,800, 1,700–1,500, and 1,200–900 cm^−1^ reflecting the spectral vibrations of different components, respectively. The PCA was performed using the second derivative spectra.

### Scanning electron microscopy and energy dispersive X-ray spectroscopy

The adsorbed copper ions on the surfaces of *Spirulina* particles were measured using SEM/energy dispersive X-ray spectroscopy (EDS) model ZEISS EVO18 equipment. The analysis was made using 20 kV scanning voltages followed by the surface of the sample sprayed with gold using an evaporate-to-vacuum equipment to make the surface conductive.

## Results and discussion

### Physiological characteristics of different *Spirulina* strains

Table 1 shows the physiological characteristics of the different *Spirulina* strains under normal light irradiation conditions for 5 days. The *Spirulina* strains clearly have different appearances giving various morphologies such as the spiral number (5–12), width (24–32 μm), and length (159–543 μm). In addition, the relative growth rate (0.18–0.26) of various strains of *Spirulina* is also distinct. Especially, *Spirulina* strains show different carbohydrate contents varying from 9.75 to 25.45%. In addition, these results indicate that all these algal strains exhibit large heterogeneity in their physiological characteristics.

**Table 1:** Physiological characteristics of *Spirulina* strains.

**Strains**	**Spiral number**	**Width (**μ**m)**	**Length (**μ**m)**	**Growth rate**	**Carbohydrate (% of DW)**
AH53	8–10	28.66 ± 2.89	331.6 ± 48.26	0.240 ± 0.036	20.94 ± 0.09
AN8	7–12	24.52 ± 1.1	333.8 ± 86.52	0.206 ± 0.019	9.75 ± 0.32
HS7	6–7	31.24 ± 1.88	543 ± 129.5	0.194 ± 0.034	12.96 ± 0.64
RH44	6–10	24.96 ± 3.09	471.2 ± 88.55	0.232 ± 0.020	25.45 ± 0.92
RN3	8–9	27.2 ± 2.69	345.8 ± 23.85	0.247 ± 0.010	13.22 ± 0,77
RZ22	8–13	32.32 ± 1.3	159 ± 44.49	0.252 ± 0.046	19.15 ± 0.41
WS47	5–6	25.92 ± 2.57	398.8 ± 48.69	0.264 ± 0.030	12.15 ± 0.59
XS58	6–8	32.38 ± 4.04	494 ± 118.25	0.181 ± 0.023	22.39 ± 0.68
YH46	7–9	27.28 ± 1.31	328.4 ± 68.38	0.180 ± 0.010	10.47 ± 0.08

### Copper ions removal tests

**Figure 1:**
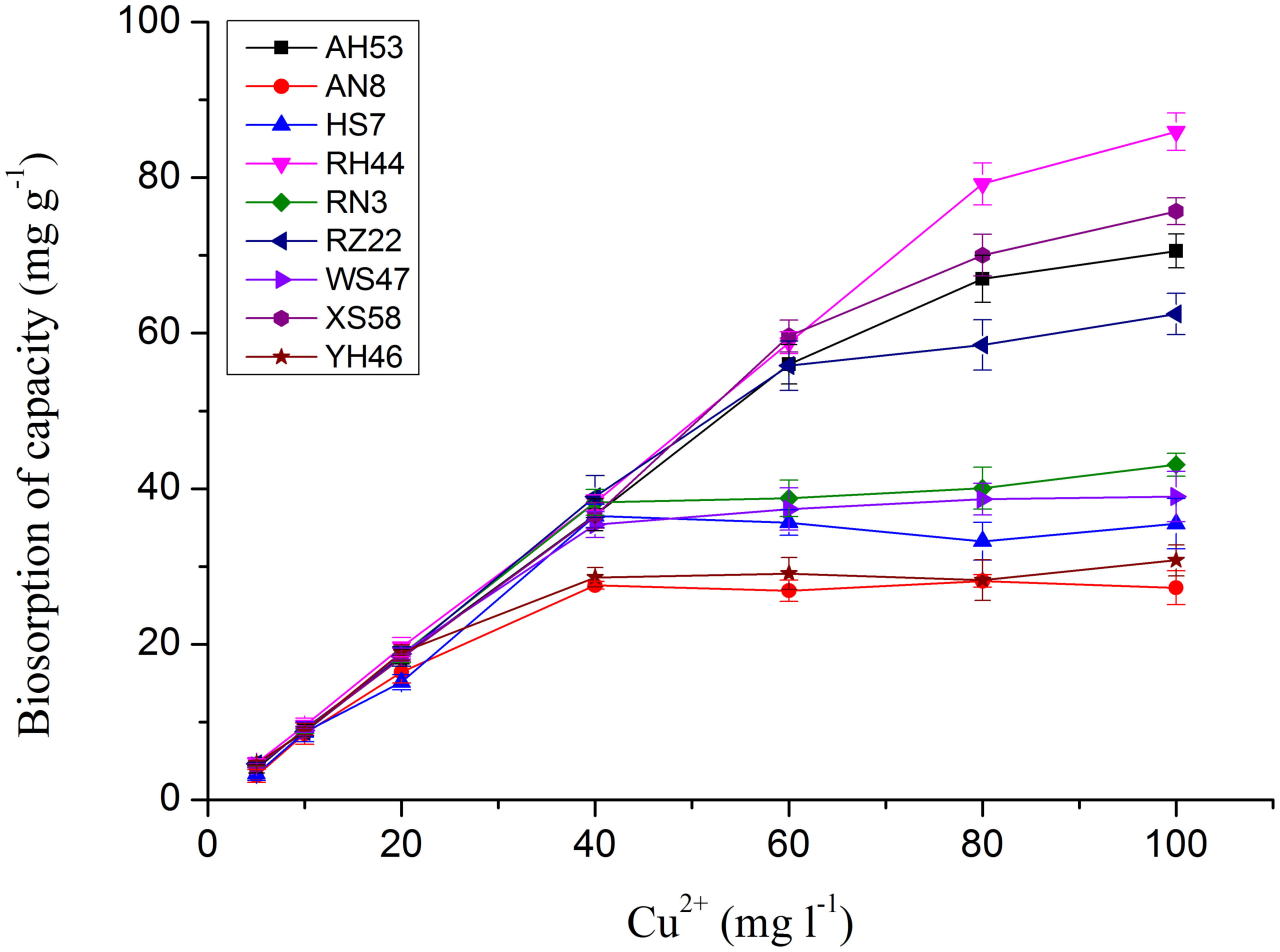
The effect of initial copper ion concentration on copper ion adsorption by *Spirulina* biomasses at the optimum pH of 6.0 for 12 h.

It has been reported that the copper adsorption increased with the raise of the pH values due to the availability of more binding sites for copper ions, and the optimum pH of 6.0 was chosen for copper ions removal since Cu(OH)_2_ precipitates above this pH value (Yang et al., 2017; Sun et al., 2019). As the copper ions began to precipitate at the initial concentration of up to 100 mg/L, experiments used to evaluate the effect of initial adsorbate doses were performed at concentrations ranging from 5 to 100 mg/L. As shown in Figure 1, for each *Spirulina* strain, the uptake of toxic ions enhanced with the raise of initial metal ion doses. At the initial metal ion dose of 100 mg/L, the maximum adsorption capacity of each algal biomass was achieved.

The copper uptake by *Spirulina* biomass increased slowly at the initial copper ion concentration from 5 to 60 mg/L. However, the enhancement of the total toxic ion removal sharpens when the initial concentration continued to be raised. Abrupt increases of adsorption capacity in the strains of RH44, XS58, AH53, and RZ22 were observed at the initial metal ion concentrations ranging from 60 to 100 mg/L which were two times higher than that in the other *Spirulina* strains. The chemical results revealed that there were different copper ions removal efficiencies in various algal strains. In order to further scrutinize the removal difference, in the following sections, FTIR spectral analysis of the components in algal cells was performed.

### Preprocessing method evaluation

**Figure 2:**
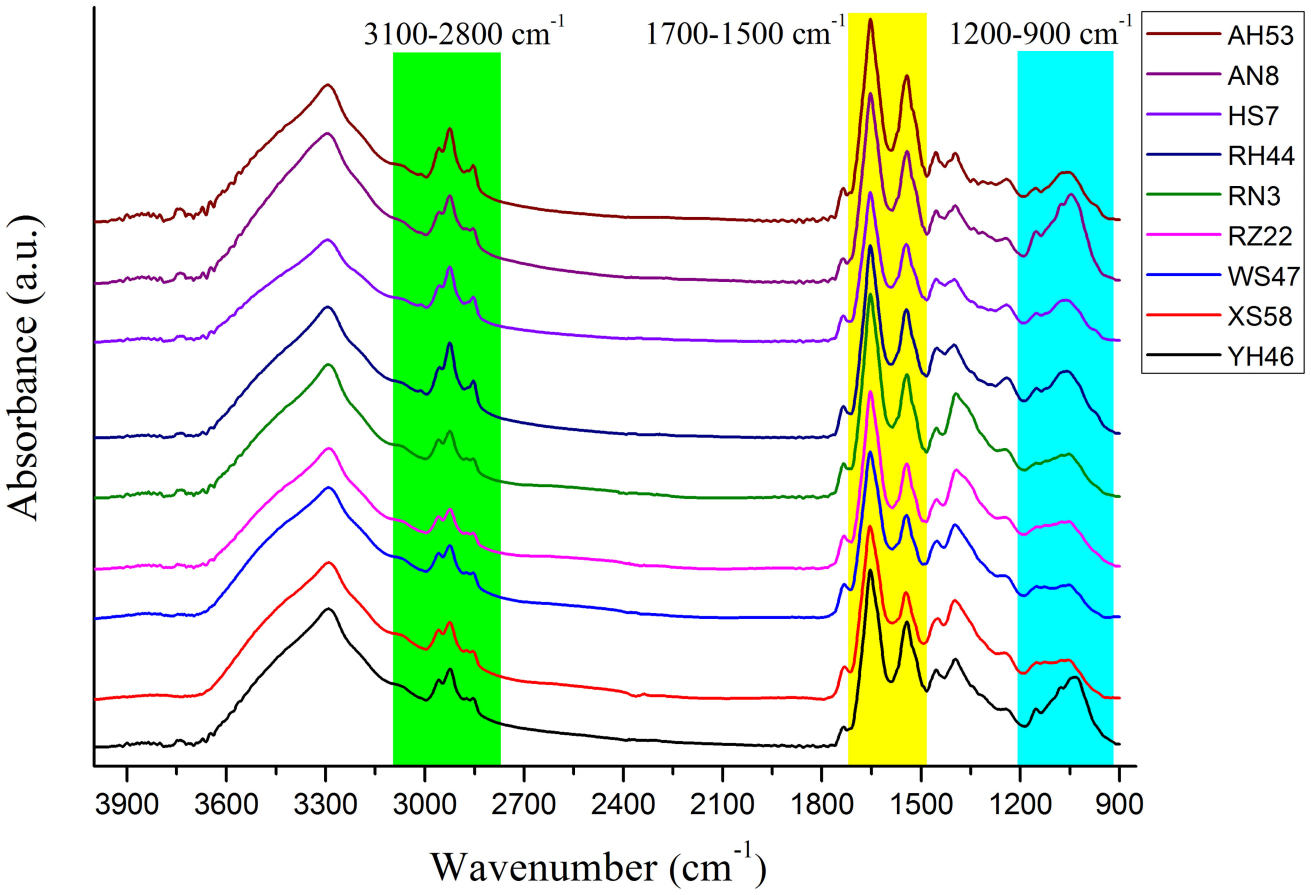
The average FTIR raw spectra and the truncated spectra for different *Spirulina* strains.

Figure 2 presents the analysis of spectra from individual *Spirulina* strains. The major bands can be assigned to the cellular constitutes such as carotenoids, lipids, polysaccharides, and proteins as outlined in Table 2 (Fuentes-Grünewald et al., 2015; Xin et al., 2019). The absorbance peak at 3,010 cm^−1^ represents the vibrational mode of CH=CH in lipids and carotenoids, while the bands at 2,955, 2,920, 2,875, and 2,850 cm^−1^ are mainly due to the asymmetric and symmetric stretching vibrations of the CH_2_ and CH_3_ in lipid and carotenoids separately, so the absorbance band observed in the range of 3,100–2,800 cm^−1^ is mainly attributed to CH stretching vibrational modes from lipids and carotenoids (Liu et al., 2020; Ahmmed et al., 2021). The FTIR spectral band at 1,740 cm^−1^ corresponds to the vibrations of C=O in lipids. The peak at 1,650 cm^−1^ is attributed to the stretch of C=O from Amide I, and the band at 1,546 cm^−1^ is assigned to the N–H stretching and C–N bending vibrations from Amide II, so the FTIR spectral region from 1,700–1,500 cm^−1^ is characterized by the amide bands in proteins. In contrast, the peaks within 1,200–900 cm^−1^ are mainly associated with C–O–C stretching from polysaccharides (Kavitha et al., 2021; Zhang et al., 2022). It can be seen that the cellular constitutes vary in different algal strains which can be identified by the infrared spectra. Therefore, the *Spirulina* strains can be discriminated by their corresponding spectral variances.

**Table 2:** Assignments of the main absorption bands in the FTIR spectrum of *Spirulina*.

**Absorption band (cm** ^−1^ **)**	**Assignment**
~3,010	trans-CH=CH-
~2,955	υ_asym_ CH_3_ in lipids
~2,920	υ_asym_ CH_2_ in lipids
~2,875	υ_sym_ CH_3_ in lipids
~2,850	υ_sym_ CH_2_ in lipids
~1,740	C=O stretching in lipids and fatty acids
~1,650	Amide I: C=O vibration
~1,546	Amide II: N–H and C–N vibration
~1,455	CH_2_/CH_3_ in lipids and proteins
~1,240	υ_asym_ ${\text{PO}}_{2}^{-}$in nucleic acids or phospholipids
~1,200–900	υ(C–O–C) of polysaccharides

*υ_asym_, asymmetric stretch; υ_sym_, symmetric stretch*.

**Figure 3:**
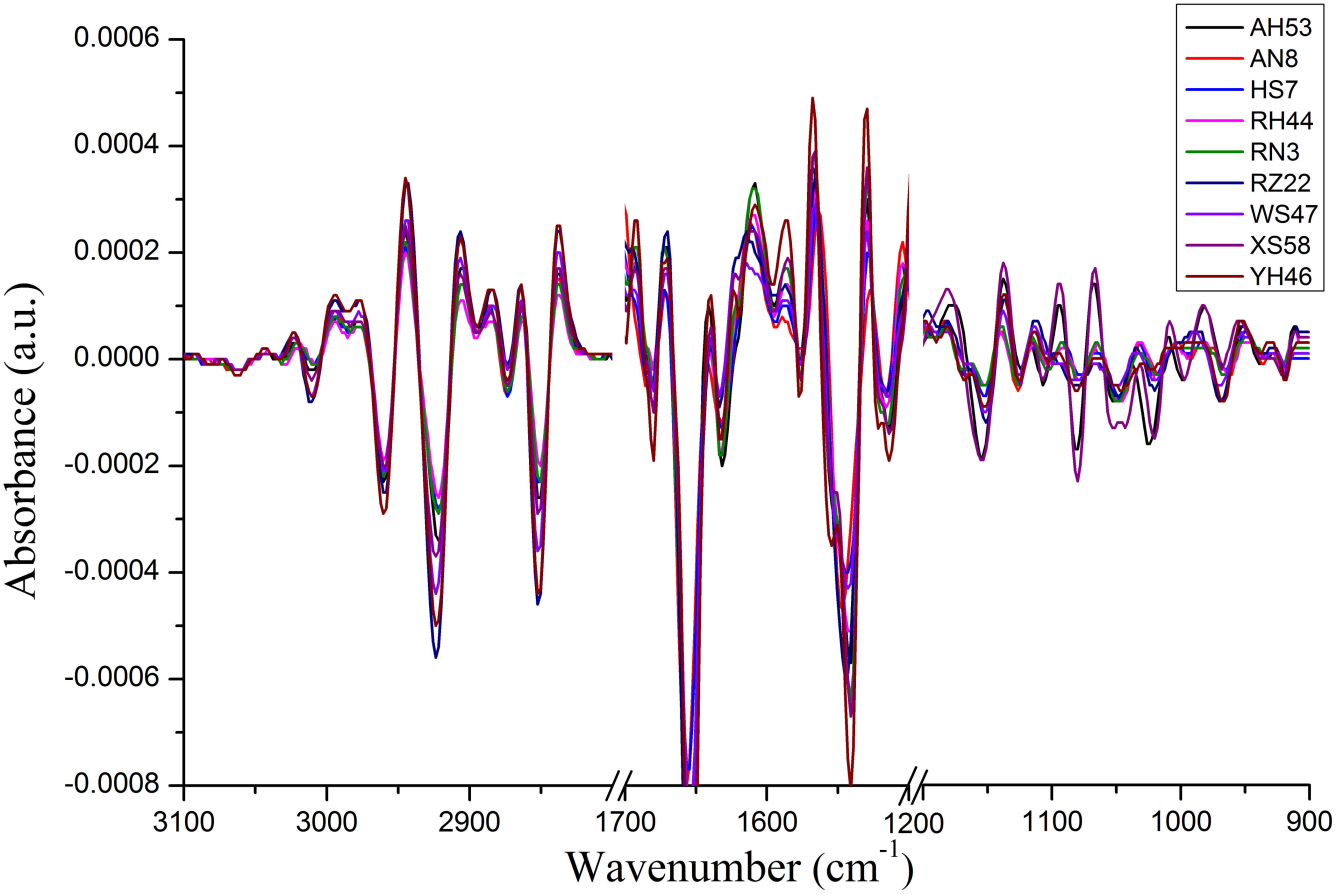
The second derivative FTIR spectra collected from each *Spirulina* strain.

For the FTIR spectra of each *Spirulina* strain, the differences manifest themselves mostly in changes of lipids/carotenoids, proteins, and polysaccharides, and these bands are located at the regions of 3,100–2,800, 1,700–1,500, and 1,200–900 cm^−1^. In addition, these changes can be much more evident in the second derivative spectra as presented in Figure 3, such as the spectral frequency shifts. In addition, it can reveal the good homogeneity of the FTIR spectra from the various *Spirulina* strains based on the spectral data pretreatment using the calculation of the second derivative.

### Algal strains classification by PCA-based FTIR analysis

**Figure 4:**
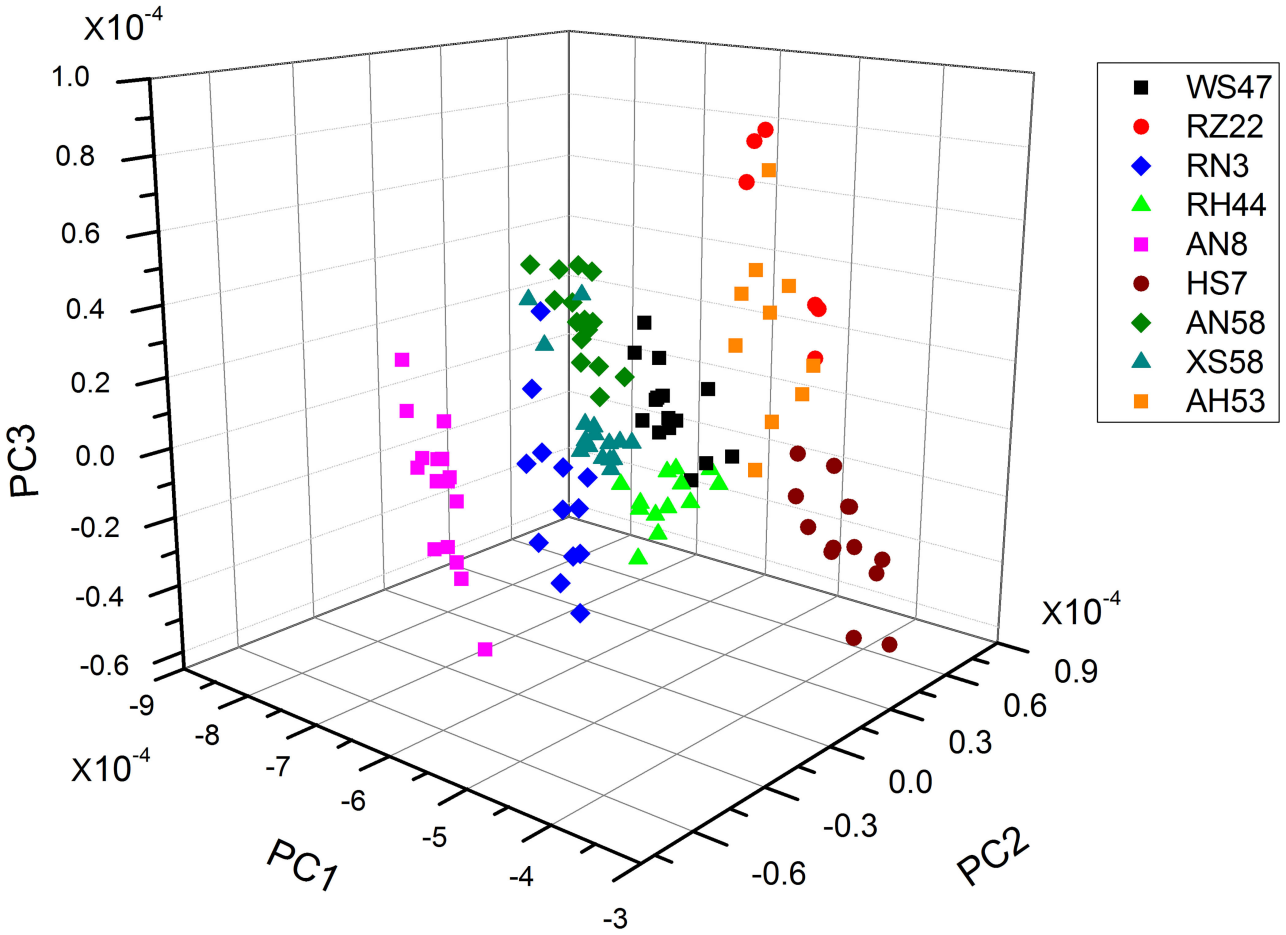
PCA results of the carotenoids and lipids region (ca. 3,100–2,800 cm^−1^).

**Figure 5:**
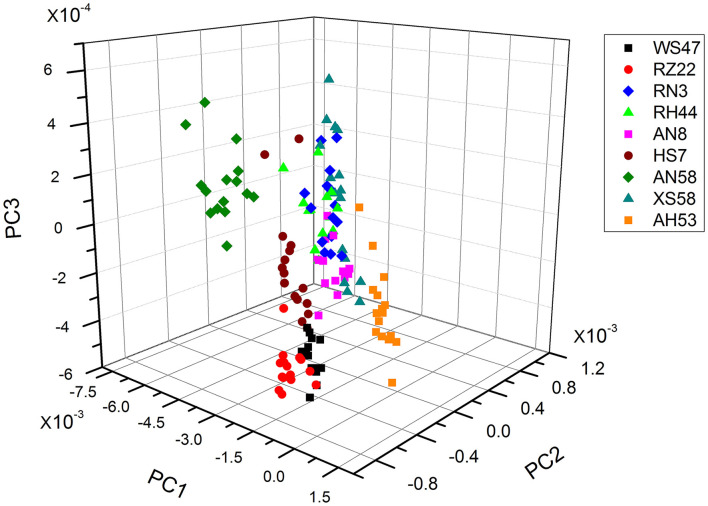
PCA results of the carotenoids and lipids region (ca. 1,700–1,500 cm^−1^).

**Figure 6:**
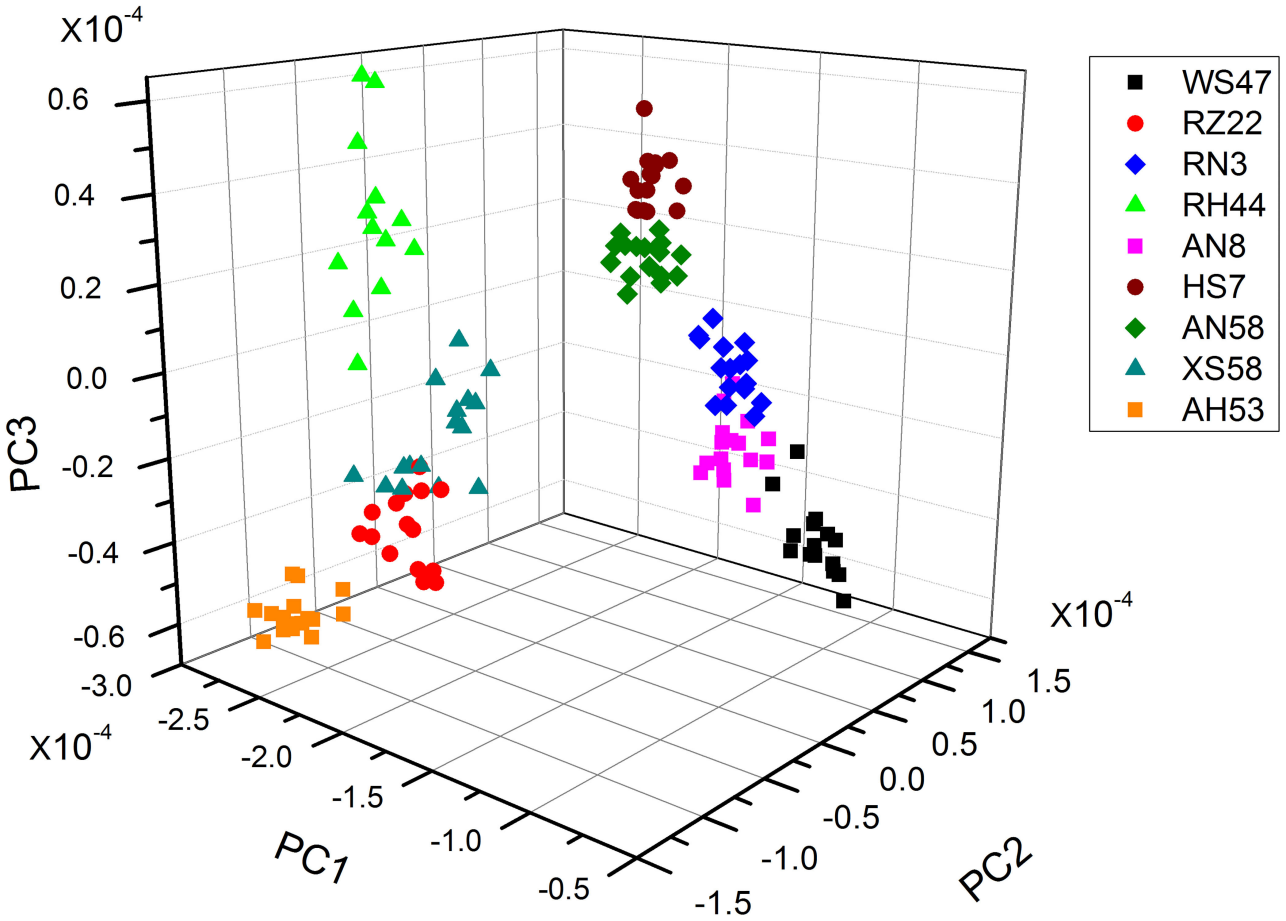
PCA results of the proteins and polysaccharides region (ca. 1,200–900 cm^−1^).

As the individual spectra are visualized similarly, a method of multivariate analysis for a meaningful interpretation is further required. For the classification of spectral data, a common multivariate statistical method (PCA) was employed to discriminate various algal strains based on their spectral characterizations. A score plot of the *Spirulina* cellular populations is demonstrated in Figures 4–6. By viewing the score plots of spectra ranges at 3,100–2,800 and 1,700–1,500 cm^−1^ in Figures 4, 5, they do not show distinct clusters among different algal strains, indicating that there are no distinct spectral differences in the region for lipids, carotenoids, and proteins. However, as shown in Figure 6, the algal strains for high copper adsorption capacity (RH44, XS58, AH53, and RZ22) can be easily differentiated based on the spectral variations at a window range of 1,200–900 cm^−1^ which match very well with the carbohydrate levels in various *Spirulina* strains, as shown in Table 1. Moreover, the clusters achieved from the spectral analysis are well consistent with the results of copper ion removal obtained by chemical assay, so it appears fairly good in the evaluation and prediction of the copper removal efficiency in different *Spirulina* strains by FTIR.

Actually, it has been widely recognized and confirmed that the metal cations removal from the aqueous solutions is attributed to the complex formation between heavy metal ions and the functional groups present on the cell surface. For instance, Wei and co-workers proved that proteins, humic acids, and polysaccharides exhibited a relatively high adsorption capacity for Zn^2+^, Cu^2+^, and Cd^2+^ (Wei et al., 2019). Sahmoune (2018) hypothesized that the uptake of heavy metals by *Streptomyces rimosus* occurs due to the interaction between metal ions and carboxyl groups from the cell walls. In contrast, the results obtained in this study suggested that the polysaccharides were key chemicals responsible for the diversity of copper adsorption capacity in different algal strains. In addition, FTIR spectroscopy facilitated the screening of *Spirulina* strains for high copper adsorption capacity based on the spectral features of algal constitutes.

### Scanning electron microscopy

**Figure 7:**
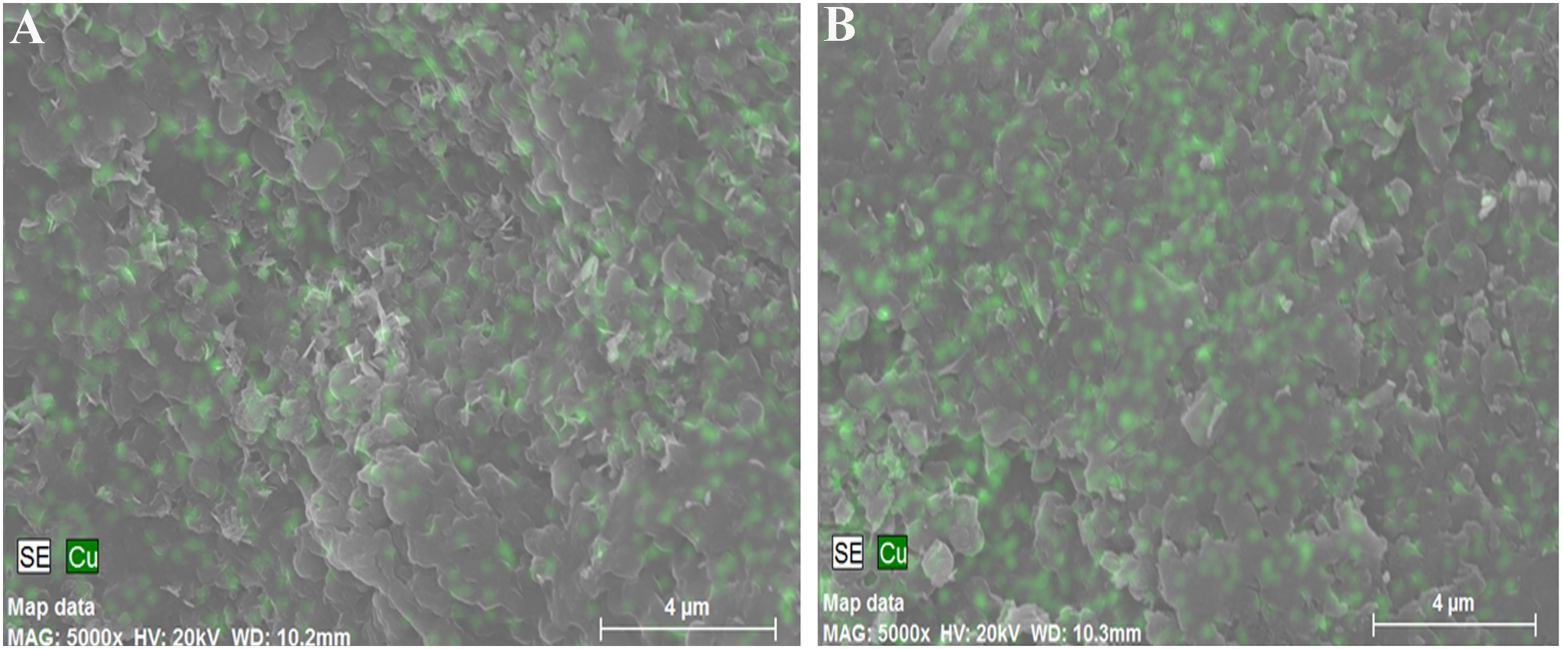
Scanning electron micrograph of *Spirulina* strains AN8 **(A)** and RH44 **(B)**.

The surfaces of two different *Spirulina* strains were analyzed by SEM after the adsorption of copper ions, as shown in Figure 7. It can be seen that the morphological sizes of particles for *Spirulina* strains are very uniform followed by ground to powders and sieved through a sieve. In addition, the biochemical composition of *Spirulina* particles can be analyzed by EDS. It is obvious that biosorbent incubated with CuSO_4_ solution contains abundant copper element, while its content in the strain RH44 is relatively higher than that in the strain AN8, which agrees well with the experimental data of copper ion removal in Figure 1.

## Conclusion

We have investigated the physiological characteristics of several *Spirulina* strains and tested their maximum adsorption capacity for copper ions. In particular, the functional groups in the algal cells have also been measured by FTIR spectroscopy. In this study, we have found that FTIR spectroscopy is a convenient physicochemical tool to scrutinize biomaterials and it is capable of assessing various constituents, including carotenoids, lipids, proteins, and polysaccharides. To the best of our knowledge, this is the first attempt to demonstrate that FTIR is a powerful tool to discriminate the maximum adsorption capacity in various *Spirulina* strains with the assistance of mathematical algorithm (PCA). In addition, this technique may be further developed to rapidly seek the potential algal strains with high adsorption capacities for removing heavy metal ions from industrial effluents.

## Data availability statement

The raw data supporting the conclusions of this article will be made available by the authors, without undue reservation.

## Author contributions

JL contributed to conceptualization, data curation, formal analysis, investigation, validation, and writing the original draft. CZ contributed to conceptualization, validation, and investigation. ZL contributed to formal analysis, data curation, and validation. HZ contributed to offering the experimental materials and revising the manuscript. All authors reviewed the manuscript, contributed to the article, and approved the submitted version.

## Funding

This study was financially supported by the University Natural Science Research Project of Anhui Province of China (KJ2020A0060), the Qingchuang Science and Technology Support Program of Shandong Provincial College (LJRZ201906), and the Fund of Key Laboratory of Aquatic Product Processing, Ministry of Agriculture and Rural Affairs, China (NYJG201802).

## Conflict of interest

The authors declare that the research was conducted in the absence of any commercial or financial relationships that could be construed as a potential conflict of interest.

## Publisher's note

All claims expressed in this article are solely those of the authors and do not necessarily represent those of their affiliated organizations, or those of the publisher, the editors and the reviewers. Any product that may be evaluated in this article, or claim that may be made by its manufacturer, is not guaranteed or endorsed by the publisher.
